# In rheumatoid arthritis, changes in autoantibody levels reflect intensity of immunosuppression, not subsequent treatment response

**DOI:** 10.1186/s13075-019-1815-0

**Published:** 2019-01-18

**Authors:** Emma C. de Moel, Veerle F. A. M. Derksen, Leendert A. Trouw, Holger Bang, Gerard Collée, Leroy R. Lard, Sofia Ramiro, Tom W. J. Huizinga, Cornelia F. Allaart, René E. M. Toes, Diane van der Woude

**Affiliations:** 1 Leiden University Medical Center, Leiden, the Netherlands; 2 Orgentec Diagnostika GmbH, Mainz, Germany; 3 Haaglanden Medical Center Antoniushove, Leidschendam, the Netherlands; 4 Zuyderland Medical Center, Heerlen, the Netherlands

**Keywords:** Rheumatoid arthritis, Autoantibodies, Ant-CCP2, Rheumatoid factor, Anti-carbamylated protein antibodies, Disease activity, Immunosuppression

## Abstract

**Background:**

Rheumatoid arthritis (RA) is characterized by the presence of autoantibodies like rheumatoid factor (RF), anti-cyclic citrullinated peptide-2 (anti-CCP2), and anti-carbamylated protein (anti-CarP) antibodies. It is currently unclear whether changes in autoantibody levels are associated with disease activity/treatment outcomes and whether they are modified by treatment intensity. Therefore, we determined longitudinal changes in RA-autoantibody levels, the association between these changes and activity score (DAS) and treatment outcomes, and the effect of intensity of immunosuppressive treatment on levels.

**Methods:**

In 381 seropositive RA patients from the IMPROVED study, we measured IgG, IgM, and IgA of anti-CCP2 and anti-CarP; IgM and IgA of RF; and IgG against four citrullinated and two acetylated peptides at 4-month intervals over the first year of treatment. Following initial prednisone and methotrexate (MTX), treatment was changed every 4 months aiming for DAS < 1.6. We investigated changes in autoantibody levels following treatment escalation versus tapering, and the association of levels with DAS over time, EULAR response, and drug-free remission (DFR) ≥ 1 year.

**Results:**

For all 14 autoantibodies, levels decreased from 0 to 4 months and then rose until 12 months. Following treatment escalation, autoantibody levels dropped markedly, while they rose following tapering: RF IgM levels, a representative autoantibody, dropped 10% after restarting prednisone and rose 15% aU/mL after tapering MTX (*p* < 0.0001). There was no association between autoantibody levels and DAS over time or EULAR response. Greater relative changes between 0 and 12 months did not predict DFR (0–12-month relative change RF IgM, − 39% for no DFR (*n* = 126) and − 16% for DFR (*n* = 18)).

**Conclusions:**

Changes in RA-autoantibody levels are not associated with DAS or long-term treatment response, but reflect intensity of immunosuppression. This suggests that autoantibody levels are modifiable by current therapies, but that modifying levels is in itself of limited clinical relevance.

**Trial registration:**

ISRCTN11916566. Registered on 7 November 2006

**Electronic supplementary material:**

The online version of this article (10.1186/s13075-019-1815-0) contains supplementary material, which is available to authorized users.

## Introduction

Rheumatoid arthritis (RA) is a chronic inflammatory joint disease characterized by the presence of autoantibodies. Rheumatoid factor (RF) and anti-cyclic citrullinated peptide-2 (anti-CCP2) are the most well-known of these, but other autoantibody systems such as anti-carbamylated protein (anti-CarP) and anti-acetylated peptide antibodies have also been identified [1, 2]. Autoantibody-positive patients have a worse prognosis, more radiographic damage, and a lower chance of achieving drug-free remission [3, 4].

Serum concentrations of these autoantibodies may change over time. Given the link between autoantibodies and disease severity and the value of measuring autoantibodies in other autoimmune diseases, these serological changes in RA may hold promise as an accessible biomarker for the future disease course. A substantial drop in autoantibody levels may, for example, be hypothesized to precede successful drug-free remission. However, studies documenting the relationship between fluctuations in autoantibodies and disease activity have been conflicting [5–7]. Importantly, most of these studies did not account for factors like intensity of immunosuppressive treatment, which likely influences both level changes and disease activity. It is unknown whether changes in autoantibody levels reflect immunosuppressive therapy or whether changes are indicative of future disease course. Furthermore, most studies investigated a limited number of autoantibodies and did not take “newer” autoantibodies into account, such as anti-CarP, which has been described to be associated with disease activity [8].

Because of this, the clinical implications of changes in autoantibody levels remain unclear, but are potentially relevant for two reasons. First, if autoantibody levels are a marker of future disease activity, it may be useful to measure pre-treatment values or monitor level changes over time. Second, understanding the changes in autoantibody levels and their association with both immunosuppression and disease activity might shed new light on mechanisms underlying the B cell autoimmune response in RA and its role in disease persistence. To that end, we longitudinally characterized changes in RA-associated autoantibody levels over time and investigated whether levels are affected by intensity of immunosuppressive treatment, how they associate with disease activity over time, and whether level changes associate with both short-term and long-term treatment outcomes.

## Methods

### Study design, patient selection, and outcomes

The Induction therapy with Methotrexate and Prednisone in Rheumatoid Or Very Early arthritic Disease (IMPROVED) study is a multicenter, randomized controlled trial that enrolled 610 patients with early (< 2 years) untreated RA or undifferentiated arthritis. It was steered at disease activity score-remission (DAS44 < 1.6) and for those achieving remission, at drug-free remission (DFR), with treatment adjusted every 4 months according to whether treatment targets had been reached. Initial treatment comprised methotrexate (MTX) and high-dose prednisone [4].

Subjects selected for this study were all 381 patients fulfilling the 2010 ACR/EULAR RA criteria with serum available at least once within the first year and seropositive by routine clinical testing for anti-CCP2 IgG, RF IgM, or our in-house assay for anti-CarP IgG at baseline or at 1 year (details described in [9]). Clinical outcomes investigated were DAS, health assessment questionnaire (HAQ), EULAR response at 4 and 12 months, and long-term sustained DFR. Long-term sustained DFR was defined as disease-modifying anti-rheumatic drug (DMARD)-free remission lasting at least 1 year, starting at any time point and continuing until the last moment of that individual’s follow-up (maximum of 5 years follow-up). Radiographic progression at 1 and 5 years was assessed using the Sharp/van der Heijde Score (SHS), as previously described [4].

### Serological measurements

Enzyme-linked immunosorbent assays (ELISAs) were used as described previously [9] to measure at 4-month intervals over the first year of treatment: anti-CCP2 IgG, IgM, and IgA; RF IgM and IgA; anti-CarP IgG, IgM, and IgA; anti-citrullinated-vimentin 59–74 IgG, anti-citrullinated-fibrinogen β 36–52 and α 27–43 IgG, and anti-citrullinated-enolase 5–20 IgG (all in-house assays); and anti-acetylated lysine vimentin IgG and anti-acetylated ornithine vimentin IgG (Orgentec Diagnostika GmbH, Germany) [1]. Samples were considered positive if they fell above a cutoff of the mean arbitrary units (aU) per milliliter plus two standard deviations of 76 sera of healthy controls from the Leiden area.

Composites reflecting the number of autoantibodies present at every time point were constructed: the number of isotypes: anti-CCP2 IgG, IgM, IgA; RF IgM and IgA; anti-CarP IgG, IgM, IgA (range 1–8), and the number of IgG anti-modified peptide antibodies (AMPAs): anti-CCP2, anti-CarP, and the antibodies against citrullinated and acetylated peptides described above (range 1–8).

### Statistical analysis

Longitudinal, repeated measures data (autoantibodies, DAS, HAQ) were modeled using generalized estimating equations (GEE), which allow missing data in the outcome. A model with a Toeplitz (m-dependent) correlation structure and a standard Gaussian distribution was chosen (akin to linear regression). For the number of autoantibodies over time (count data), a negative binomial model was specified.

With repeated measurements of the autoantibody levels/number as the dependent variable, we investigated by GEE whether a certain treatment decision (4 months, no change versus escalation of treatment; 8 months: tapering versus escalation of treatment) was associated with a subsequent change in autoantibody levels/number. An interaction term of treatment decision * time was used to assess whether changes in the autoantibody levels over time were different in patients that tapered/did not change versus those that escalated immunosuppressive therapy. For comparison purposes, a normalization of the different measurement units was applied to the final model estimates (which were in aU/mL) by dividing them by the maximum of the autoantibody’s range.

The association between autoantibody levels and DAS over time was investigated using GEE, with DAS as the outcome. The same was conducted for HAQ over time. Ordinal, logistic, and linear regression was used to investigate the association of relative changes in autoantibody levels/absolute changes in number of autoantibodies with EULAR response, long-term sustained DFR, and SHS radiographic progression scores, respectively.

All models were adjusted for gender and age; clinical outcome analyses were adjusted for treatment decisions. Other covariates (i.e., disease duration, smoking, body mass index (BMI), baseline HAQ/DAS) were only included in final models if they were univariably associated with the outcomes of interest (*p* < 0.1). Holmes-Bonferroni methods were used to correct all analyses for multiple testing, assuming the same number of tests as autoantibodies investigated (14 tests).

## Results

### Autoantibody levels decrease upon initiation and escalation of immunosuppressive treatment

**Fig. 1:**
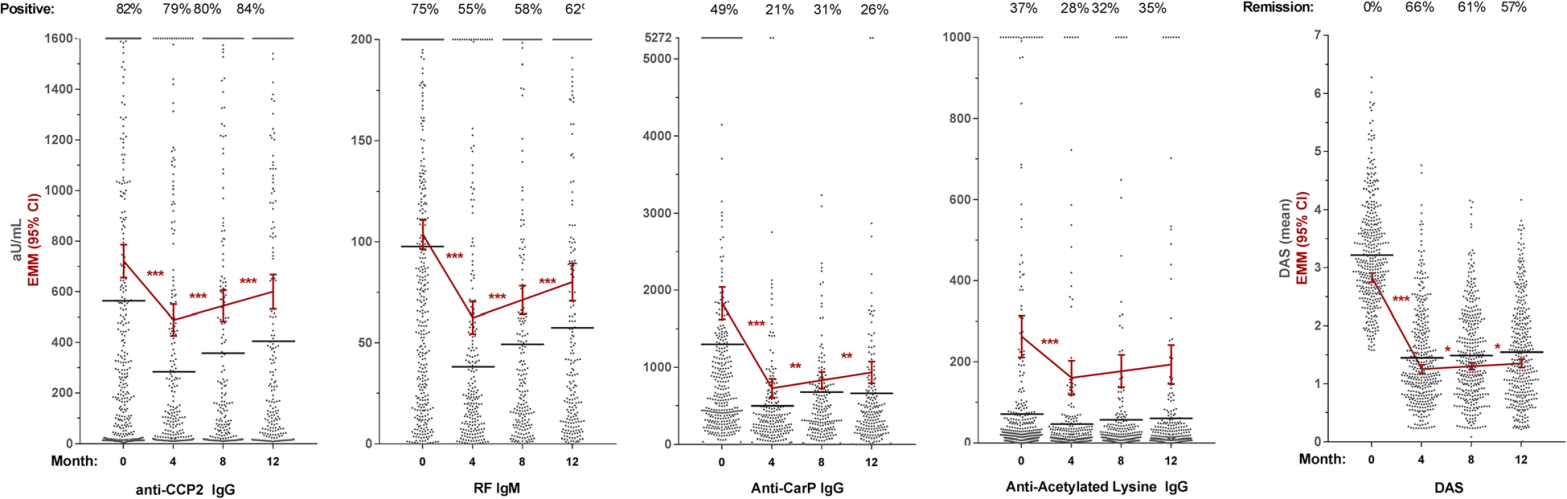
Autoantibody levels initially decrease and then steadily rise over time, paralleled by disease activity. Levels in arbitrary units (aU/mL) of four representative autoantibodies and DAS over the first year of treatment as measured in the serum of seropositive RA patients (for levels: *N* 0 months = 356; *N* 4 months = 225; *N* 8 months = 209; *N* 12 months = 212; for DAS: *N* 0 months = 381; *N* 4 months = 374; *N* 8 months = 361; *N* 12 months = 357). Patients clustered at the maximum were above the highest standard of the ELISA. Black lines indicate median level in arbitrary units per milliliter or mean DAS. Red lines indicate estimated marginal mean (EMM) arbitrary units per milliliter or DAS with 95% confidence intervals (calculated by GEE). *p* values (asterisk) refer to the change between two time points. *p < 0.05, **p < 0.01, ***p < 0.001

For all 14 autoantibodies, median levels decreased significantly between baseline and 4 months when prednisone and MTX were initiated, and then stabilized or steadily increased until 12 months, while DAS plummeted between 0 and 4 months and stayed low between 4 and 12 months (Fig. 1; Additional file 1: Figure S1 for all autoantibodies).

**Fig. 2:**
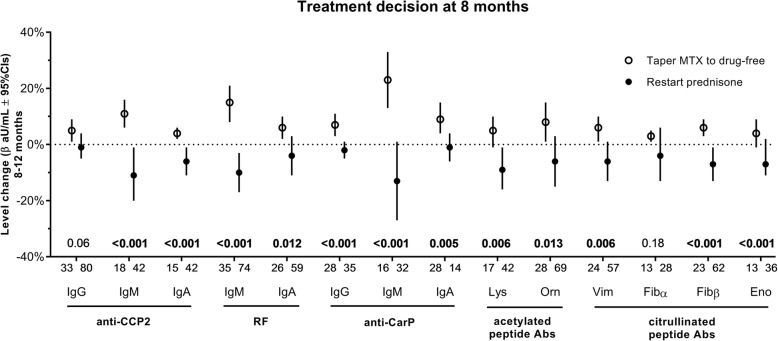
Autoantibody levels change following treatment decisions. Change in autoantibody levels (calculated by GEE) following treatment decision at 8 months, within patients that were positive for that autoantibody at least once over the first year. Depicted regression coefficients (β in aU/mL, with 95% CIs) are of the predictor *time* from a GEE model stratified for the treatment decision, and thus indicate autoantibody level changes between 8 and 12 months for that treatment decision group. Coefficients were normalized for comparison purposes by dividing by the maximum arbitrary units per milliliter of the ELISA range of each autoantibody. Models were adjusted for age, gender, smoking status, and disease duration. Bold typeface of *p* values calculated by GEE (interaction term treatment*time) indicates significance after Holmes-Bonferroni correction for multiple testing (14 tests)

Due to its design, the IMPROVED study can be used to investigate whether autoantibody levels might decrease not only upon treatment initiation, but also upon decisions regarding intensity of immunosuppression, by examining changes in autoantibodies after treatment was either tapered or escalated. We first looked at the moment in the IMPROVED study when one would expect the largest differences: at 8 months, patients that had achieved DAS-remission tapered MTX monotherapy to drug-free, while those not in remission escalated therapy by restarting prednisone (next to MTX). It was found that autoantibody levels rose between 8 and 12 months following the decision to taper MTX to drug-free and dropped if prednisone was restarted (Fig. 2). This finding was significant for 12/14 autoantibodies.

Another treatment decision was the one made at 4 months: patients that achieved remission at 4 months continued MTX monotherapy, while those that did not were randomized to one of the two treatment arms, where therapy was escalated by an addition of multiple DMARDs (prednisone, hydroxychloroquine, and sulfasalazine; arm 1) or adalimumab (arm 2). Levels consistently rose between 4 and 8 months during continued treatment with MTX while they either dropped or stayed relatively stable following treatment escalation (Additional file 1: Figure S2). At this moment, the difference in treatment intensity was less pronounced than at 8 months (i.e., no patients tapered to drug-free), as were the differences in autoantibody level changes.

The total autoantibody number changed in a manner similar to the changes in levels, both at treatment initiation (Additional file 1: Figure S1) and for the decision at 4 and at 8 months (not shown).

This indicates that autoantibody levels are responsive to immunosuppression and likely change upon decisions to escalate or taper therapy. The next question was whether these autoantibody changes are associated with treatment outcomes, and thus whether the changes in autoantibody levels in response to treatment might be clinically relevant to monitor over time.

### Autoantibody levels are not longitudinally associated with DAS

Given the way both autoantibody levels and DAS decreased upon treatment, we addressed the question whether the two are longitudinally and independently associated (Table 1). The GEE models reported in Table 1 are congruent with both a cross-sectional and a longitudinal interpretation. First, a patient that is 1 unit (aU/mL) higher in an autoantibody level is expected, at that moment, to have a higher DAS of the indicated magnitude (e.g., 0.011 DAS units per 100 aU/mL anti-CCP2 IgG). Second, a patient that increases 1 unit in an autoantibody (for any given time interval) is expected to have an *increase* in DAS of the indicated magnitude for that same time interval. Although almost all associations of autoantibodies and DAS changes were significant (confidence intervals do not cross zero), the magnitude of association was miniscule and far from clinically relevant. We conclude that there is no relevant association between autoantibody level changes and DAS changes.

**Table 1:** Association of DAS over time with autoantibody levels over time

Outcome: DAS over time	*n*	β (95% CI) in DAS units per aU/mL
Anti-CCP2 IgG (0–1600)	259	1.1 × 10^−4^ (9.3 × 10^−6^ to 2.0 × 10^−4^)
Anti-CCP2 IgM (0–1400)	129	3.5 × 10^−4^ (1.4 × 10^−4^ to 5.7 × 10^−4^)
Anti-CCP2 IgA (0–1160)	128	3.6 × 10^−4^ (3.8 × 10^−5^ to 6.7 × 10^−4^)
RF IgM (0–200)	245	1.8 × 10^−3^ (8.2 × 10^−4^ to 2.8 × 10^−3^)
RF IgA (0–200)	188	7.9 × 10^−4^ (− 8.9 × 10^−5^ to 1.7 × 10^−3^)
Anti-CarP IgG (0–5272)	149	1.1 × 10^−4^ (4.1 × 10^−5^ to 1.7 × 10^−4^)
Anti-CarP IgM (0–3650)	128	1.7 × 10^−4^ (8.9 × 10^−5^ to 2.6 × 10^−4^)
Anti-CarP IgA (0–3100)	99	2.7 × 10^−4^ (1.6 × 10^−4^ to 3.8 × 10^−4^)
Anti-acetyl-lysine IgG (0–1000)	128	2.5 × 10^−4^ (2.1 × 10^−5^ to 4.7 × 10^−4^)
Anti-acetyl-ornithine IgG (0–1000)	226	2.0 × 10^−4^ (2.4 × 10^−5^ to 3.8 × 10^−4^)
Cit-Vim IgG (0–10,000)	188	3.7 × 10^−5^ (1.2 × 10^−5^ to 6.1 × 10^−5^)
Cit-Fib α IgG (0–25,000)	88	1.6 × 10^−6^ (− 2.0 × 10^−5^ to 2.3 × 10^−5^)
Cit-Fib β IgG (0–100,000)	191	4.2 × 10^−6^ (2.2 × 10^−6^ to 6.2 × 10^−6^)
Cit-Eno IgG (0–70,000)	105	2.9 × 10^−6^ (− 8.8 × 10^−7^ to 6.8 × 10^−6^)

Generalized estimating equation of the continuous outcome DAS over the first year of treatment, within patients that were positive for that autoantibody at least once over the first year. Models were adjusted for age, gender, baseline HAQ, time, randomization arm at 4 months, and treatment decision at 8 months. Values beside the autoantibody names indicate range (in aU/mL)

Autoantibody levels and HAQ were generally not significantly associated, and if they were (significant for only 3/14 autoantibodies), the magnitude of association was similarly minute as found for DAS (not shown).

### Changes in autoantibody levels are not associated with EULAR response

**Fig. 3:**
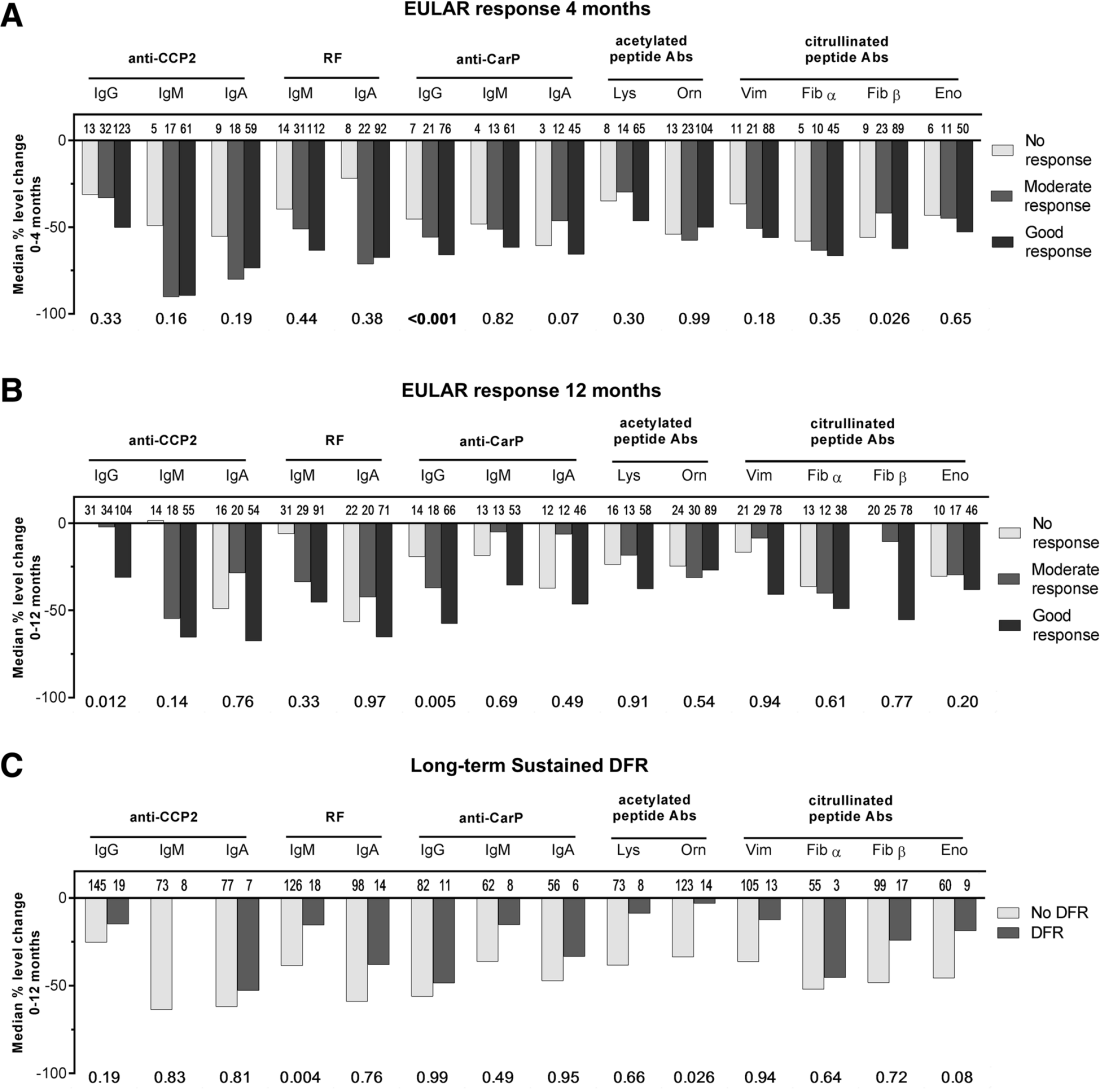
Changes in autoantibody levels do not associate with treatment outcomes. Relative change (%) in autoantibody levels (raw data) preceding **a** EULAR response at 4 months, **b** EULAR response at 12 months, and **c** long-term sustained DFR. Bold typeface of *p* values below graphs, calculated by ordinal (**a**, **b**) and logistic (**c**) regression, indicates significance after Holmes-Bonferroni correction for multiple testing (14 tests). Besides age and gender, models in **a** are adjusted for baseline DAS and BMI, in **b** are additionally adjusted for randomization arm at 4 months and treatment decision at 8 months, and in **c** are adjusted for baseline DAS, disease duration, and treatment decisions as per **b**

Most patients (264/381; 70%) had a good EULAR response at 4 months. Patients that achieved good/moderate EULAR response at 4 months had somewhat higher baseline autoantibody levels, but this small difference was not significant (data not shown). Patients that achieved good/moderate EULAR response at 4 months (or at 12 months) did not have significantly greater relative decreases in autoantibody levels between 0 and 4 months (or between 0 and 12 months, respectively) than patients with no response (Fig. 3a, b). Changes in composites of the number of isotypes or AMPAs were also not associated with EULAR response (not shown).

### Changes in autoantibody levels are not associated with long-term outcomes like sustained DFR or radiographic progression

The next outcome we wished to analyze was long-term sustained DFR, which is the closest approximation of RA cure currently available. Interestingly, relative changes in autoantibody levels or number of isotypes or AMPAs between 0 and 12 months did not significantly differ between patients that did and did not achieve DFR (Fig. 3c; composites not shown). There was also no association between autoantibody levels at 8 months and ability to achieve DFR at 12 months (not shown). This indicates that autoantibody level changes over the first year of treatment are not informative for the ability to become drug-free.

In patients with radiographs available, 19% (67/360) had radiographic progression (≥ 0.5-point change from baseline) at 1 year, and 52% (152/294) had progression at 5 years. There was no association between baseline autoantibody levels or relative changes in autoantibody levels (0–12 months) and radiographic progression points at either 1 or 5 years (not shown).

## Discussion

In RA, it is unknown whether changes in autoantibody levels are associated with disease activity and treatment outcomes, or whether levels are modified by intensity of immunosuppressive therapy. Most studies have shown that RF (IgM, IgA, and IgG) levels decrease after treatment initiation with different DMARD classes [5–7, 10, 11], while anti-CCP2 (IgG) levels decrease only marginally, rebound after decreasing, or do not decrease at all [5–7, 10–13]. Our results support this notion, as multiple forms and combinations of immunosuppressive medication in the IMPROVED led to reduction of autoantibody levels. Our results also showed that RF IgM decreases somewhat more than anti-CCP2 IgG, in line with previous reports [7].

Although the diagnostic and prognostic value of testing for autoantibody positivity in RA is well-established, reports are conflicting regarding the potential association of level fluctuations with disease activity in seropositive patients [5–7]. In the current study, there was no relevant association between autoantibody changes and disease activity. Moreover, we also found no association with functional status, treatment response, or long-term outcomes such as DFR and radiographic progression. Instead, autoantibody level changes seem to be largely a reflection of immunosuppressive therapy, rather than an indication of disease-specific clinically relevant processes. Therefore, their monitoring over time seems of limited value.

Autoantibody stability in different autoimmune diseases varies substantially, with some autoantibodies fluctuating with flares of disease, while others remain stable. These differences may be due to differences in longevity and place of residence of the autoantibody-producing cells. In RA, the synovial compartment appears to function as an inflammatory niche that promotes long-term survival of anti-citrullinated protein antibody (ACPA)-producing plasma cells [14]. ACPA-producing plasmablasts also home to the bone marrow and differentiate to long-lived plasma cells there [14, 15]. As RA disease activity subsides with anti-inflammatory treatment, the survival niches in inflamed joints may be eliminated and plasma cells residing there could be displaced and die [16]. Meanwhile, it appears plausible that bone marrow plasma cells remain unaffected by resolution of peripheral inflammation and continue to stably secrete RA-associated autoantibodies. This could explain why circulating autoantibodies show an initial decrease when treatment is initiated or intensified (elimination of joint survival niches) but are never fully eradicated (persistence of bone marrow niches), even in the absence of clinical symptoms. Whether the surviving bone marrow B cells/plasma cells contribute to the chronic pathogenic cascade remains to be determined. It is possible that such contributions may occur via pathways independent of autoantibody production, such as antigen presentation to T cells, cytokine secretion, or other immunoregulatory mechanisms. There may also exist autoantibody-specific differences in survival niches that plasma cells utilize that might explain why some autoantibodies change quite substantially upon immunosuppressive treatment while others remain more stable.

The current findings have some limitations. First, we chose not to further dilute serum samples above the highest standard of the ELISA, precluding the detection of level changes in patients with very high concentrations. However, sensitivity analyses excluding these patients yielded the same conclusions (not shown). Second, it is possible that the autoantibody level decreases seen between 0 and 4 months in all patients and between 8 and 12 months for those with treatment intensification are primarily due to the effect of prednisone, as prednisone has been shown to decrease total circulating immunoglobulin levels, especially IgG [17]. The design of this study did not allow us to investigate whether this was the case. Thirdly, we do not have serological data in the timeframe between 12 months and the long-term outcomes investigated. It is possible that antibody changes closer to the outcome are more relevant than those over the first year of treatment. Fourth, only the effect of conventional DMARDs and anti-TNF has been investigated. It may be that DMARDs with other modes of action, such as rituximab, have different effects on autoantibody levels. It is also possible that the same agents applied during a disease state may associate with different level changes than demonstrated here, as our results only apply to early RA. Finally, we recognize that due to the limited radiographic progression that occurred in the IMPROVED study, we lacked sensitivity for finding a relationship with autoantibody levels.

Strengths of this study include the extensive array of autoantibodies measured and the longitudinal nature of the analyses, which allowed analysis of absolute changes in 14 different autoantibodies spanning four autoantibody families in 4-month intervals over the first year of treatment while accounting for missing serum. The nature of the IMPROVED trial and its long follow-up also allowed us to investigate multiple short-term and long-term outcomes not previously linked to autoantibody changes, as well as the effect of immunosuppression on level changes.

## Conclusions

We conclude that in early RA, changes in autoantibody levels do not associate DAS over time or with treatment response. Instead, autoantibody level changes seem to be a reflection of treatment intensity. Together, these results suggest that autoantibody levels change over time and are modifiable by commonly used DMARDs, but that autoantibody level changes are in itself of limited clinical relevance and not useful to monitor over time.

## Additional file

Additional file 1: **Figure S1.** Levels (aU/mL), number of autoantibodies, and DAS over 1st year of treatment. **Figure S2.** Autoantibody levels change following treatment decision at 4 months. (DOCX 2424 kb)

## Abbreviations

ACPA: Anti-citrullinated protein antibody
AMPA: Anti-modified protein antibodies
Anti-CarP: Anti-carbamylated
Anti-CCP2: Anti-citrullinated protein 2
BMI: Body mass index
DAS: Disease activity score
DFR: Drug-free remission
DMARD: Disease-modifying anti-rheumatic drug
ELISA: Enzyme-linked immunosorbent assay
GEE: Generalized estimating equations
HAQ: Health Assessment Questionnaire
IMPROVED: Induction therapy with Methotrexate and Prednisone in Rheumatoid Or Very Early arthritic Disease
MTX: Methotrexate
RA: Rheumatoid arthritis
RF: Rheumatoid factor
SHS: Sharp/van der Heijde Score
