# Supplementary material for: In rheumatoid arthritis, changes in autoantibody levels reflect intensity of immunosuppression, not subsequent treatment response
Source: Arthritis Res Ther. 2019 Jan 18;21:28. doi: 10.1186/s13075-019-1815-0 (PMC6339446; doi:10.1186/s13075-019-1815-0)
Supplement: Supplementary file 1 — Figure S1. Levels (aU/mL), number of autoantibodies, and DAS over 1st year of treatment. Figure S2. Autoantibody levels change following treatment decision at 4 months. (DOCX 2424 kb) [file 13075_2019_1815_MOESM1_ESM.docx]

| **Figure S1: Levels (aU/mL), number of autoantibodies, and DAS over 1^st^ year of treatment.** Autoantibody data is from serum of seropositive RA patients by ELISA (N 0 months=356; N 4 months=225; N 8 months=209; N 12 months=212). For levels, patients clustered at the maximum were above the highest standard of the ELISA. Black and red lines respectively indicate median aU/mL and estimated marginal mean (EMM) aU/mL with 95% confidence intervals (calculated by GEE) in patients that were positive for that autoantibody at least once. Box plots indicate median, interquartile range, and 10th and 90th percentiles. P-values (asterisk) refer to the level change between two time-points. *p<0.05 **p<0.01 ***p<0.001 Number of isotypes based on anti-CCP2 IgG, IgM, IgA; RF IgM, IgA; and anti-CarP IgG, IgM, IgA; number of AMPAs based on anti-CCP2 IgG, anti-CarP IgG, anti-citrullinated-vimentin 59-74 IgG, anti-citrullinated-fibrinogen β 36-52 IgG and α 27-43 IgG, anti-citrullinated-enolase 5-20 IgG, anti-acetylated-lysine IgG, and anti-acetylated-ornithine IgG. |
| --- |
| 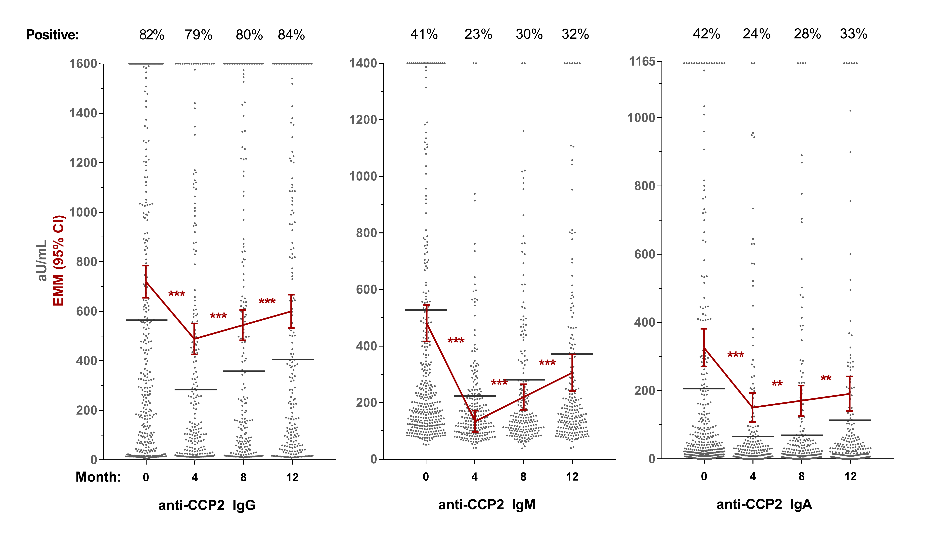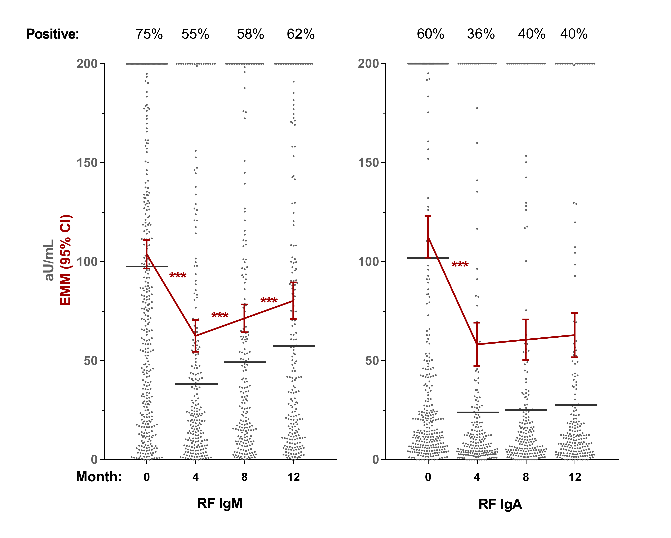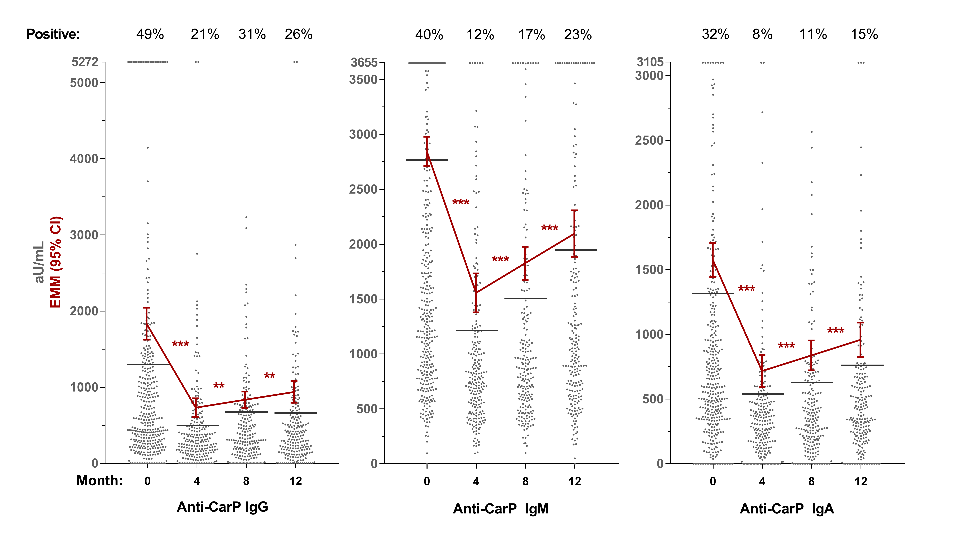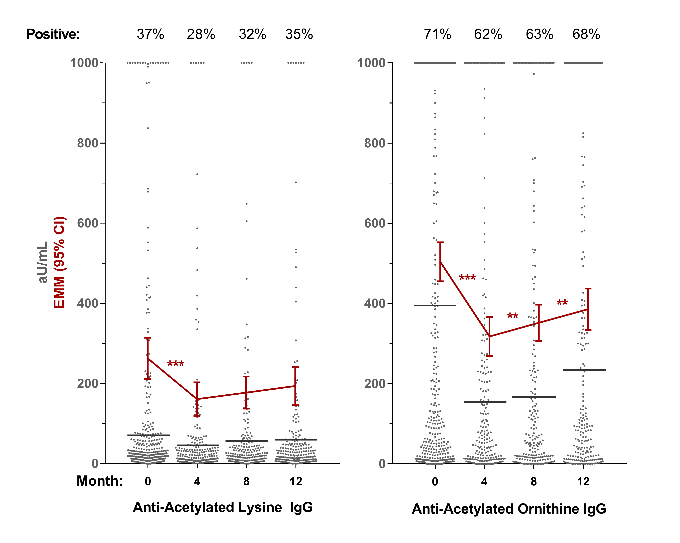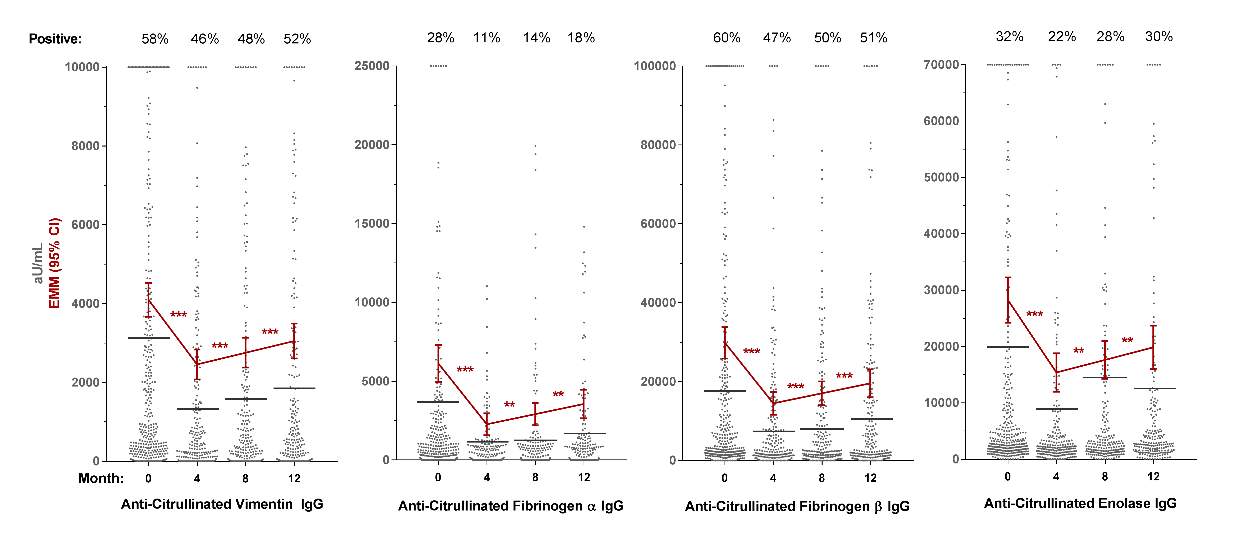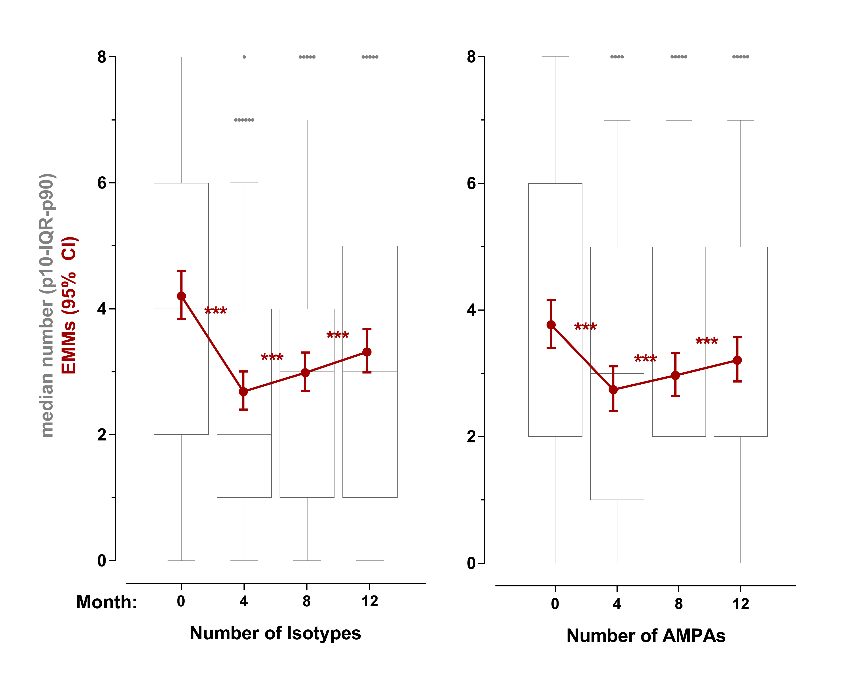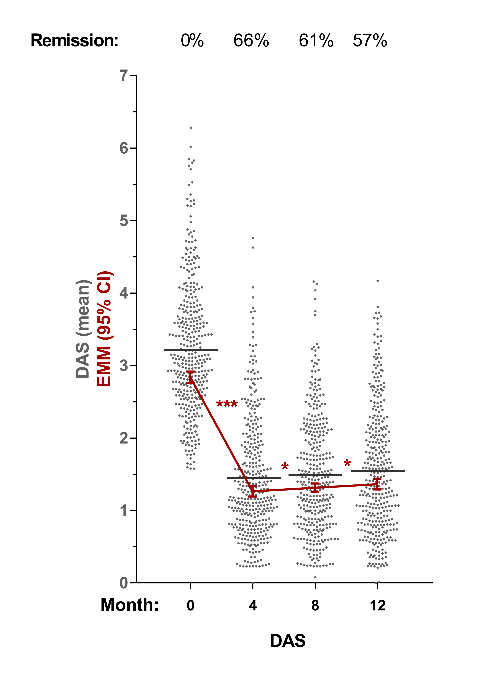 |

| **Figure S2**: **Autoantibody levels change following treatment decision at 4 months.** Change in autoantibody levels (calculated by GEE) following treatment decision at 4 months, within patients that were positive for that autoantibody at least once over the 1^st^ year. Escalation of treatment by randomization comprised addition of prednisone, hydroxychloroquine, and sulfasalazine (Arm 1) or of adalimumab (Arm 2) to MTX monotherapy. Depicted regression coefficients (β in aU/mL, with 95% CIs) are of the predictor *time* from a GEE-model stratified for the treatment decision, and thus indicate autoantibody level changes between 8-12 months for that treatment decision group. Coefficients were normalized for comparison purposes by dividing by the maximum aU/mL of the ELISA range. GEEs were adjusted for age, gender, smoking status, and disease duration. Bold typeface of p-values calculated by GEE (interaction term *treatment*time)* indicates significance after Holmes-Bonferroni correction for multiple testing (14 tests).   |
| --- |
